# Optimization of *Agrobacterium* mediated genetic transformation of cotyledonary node explants of *Vigna radiata*

**DOI:** 10.1186/2193-1801-1-59

**Published:** 2012-12-10

**Authors:** Sushil Kumar Yadav, Sweety Katikala, Varalaxmi Yellisetty, Annapurna Kannepalle, Jyothi Lakshmi Narayana, Vanaja Maddi, Maheswari Mandapaka, Arun Kumar Shanker, Venkateswarlu Bandi, Kirti Pulugurtha Bharadwaja

**Affiliations:** 1 Central Research Institute for Dryland Agriculture, Santoshnagar, Hyderabad, 500 059 India; 2 Division of Microbiology, Indian Agricultural Research Institute, New Delhi, 110 012 India; 3 Department of Life Sciences, University of Hyderabad, Hyderabad, 500 046 India

**Keywords:** *Agrobacterium* mediated transformation, Annexin, Double cotyledonary node, *Vigna radiata*

## Abstract

A reproducible and highly efficient protocol for genetic transformation mediated by *Agrobacterium* has been established for greengram (*Vigna radiata* L. Wilczek). Double cotyledonary node (DCN) explants were inoculated with *Agrobacterium tumefaciens* strain LBA 4404 harboring a binary vector pCAMBIA 2301 containing neomycin phosphotransferase (*npt II*) gene as selectable marker, β-glucuronidase (GUS) as a reporter (*uidA*) gene and *annexin 1 bj* gene. Important parameters like optical density of *Agrobacterium* culture, culture quantity, infection medium, infection and co-cultivation time and acetosyringone concentration were standardized to optimize the transformation frequency. Kanamycin at a concentration of 100 mg/l was used to select transformed cells. Transient and stable GUS expressions were studied in transformed explants and regenerated putative plants, respectively. Transformed shoot were produced on regeneration medium containing 100 mg/l kanamycin and 250 mg/l cefotaxime and rooted on ½ MS medium. Transient and constitutive GUS expression was observed in DCN explants and different tissues of T_0_ and T_1_ plants. Rooted T_0_ and T_1_ shoots confirming Polymerase Chain Reaction (PCR) positive for *npt II* and *annexin 1bj* genes were taken to maturity to collect the seeds. Integration of *annexin* gene into the greengram genome was confirmed by Southern blotting.

## Background

Grain legumes constitute an important dietary constituent for humans and animals. They associate with nitrogen fixing bacteria and play an important role in low input agricultural production systems; particularly small and marginal farm holdings. Greengram (*Vigna radiata* L. Wilczek) is an important grain legume grown widely in Southeast Asia, Africa, South Africa and Australia. The crop is grown mainly as a source of vegetable protein for its high protein content (about 25%), which makes it as an excellent supplement to cereal diets. The cultivation of this crop is gaining more popularity by virtue of its early maturity, nutritional value and easy digestibility. In India, it is cultivated mainly under limited and erratic rainfall conditions and on marginal and sub-marginal lands where numerous biotic and abiotic stresses limit its productivity (Jaiwal and Gulati 1995; Jaiwal and Singh 2003). Conventional breeding for enhancing biotic and abiotic stress tolerance in crop plants has several constraints and since the available genetic variability is low, transfer of alien genes of proven value offer possible viable option for crop improvement. Legumes in general are recalcitrant to tissue culture and are highly genotype specific (Somers et al. 2003).

Reproducible and efficient protocols for shoot regeneration have been established for greengram (Amutha et al. 2006; Kaviraj et al. 2006; Mahalaxmi et al. 2006; Mundhara and Rashid et al. 2006; Vijayan et al. 2006; Yadav et al. 2010a& 2010b). The immense potential of biotechnological tools for improving against biotic and abiotic stresses can be realized by supplementing the breeding programmes through introduction of alien genes of recognized relevance into elite germplasm of crop plants (Chandra and Pental, 2003; Somers et al. 2003; Popelka et al. 2004; Dita et al. 2006; Eapen 2008). An efficient regeneration and transformation protocol will be the key to success of genetic transformation. Though there are reports claiming successful transformation, owing to their highly recalcitrant nature in culture and very low frequency of regeneration especially after transformation, progress in development of transgenics for various legumes has been very slow (Chandra and Pental, 2003; Somers et al. 2003; Popelka et al. 2004; Dita et al. 2006). In the present study, we describe a reproducible and efficient *Agrobacterium* mediated genetic transformation protocol for greengram using double cotyledonary node (DCN) explants derived from three day old seedlings with binary vector pCAMBIA 2301 containing *annexin* gene. Ectopic expression of *annexin* has been shown to improve tolerance to various biotic and abiotic stresses in tobacco plants (Jami et al. 2008).

Annexins are Ca^2+^ and phospho-lipid binding proteins forming an evolutionary conserved multi-gene family expressed throughout plant kingdom. Annexins play a critical role in plant cell from regulation of Ca^2+^ dependent biochemical signalling processes to phospho-lipid metabolism. Gene expression of annexins in plants appears to be regulated by developmental and environmental signals and is known to be regulated by Ca^2+^ in stimulus response coupling in many plant cell–signalling pathways. Plant annexins from *Medicago sativa* and *Arabidopsis thaliana* have been implicated in oxidative stress response. We hypothesis that incorporation of annexin transgene will contribute to better tolerance to oxidative stress as the crop is predominantly grown in conditions which generate ROS. Annexin 1 from *Brassica juncea* was used in the present study.

## Results and discussion

Earlier, hypocotyl and primary leaves excised from 2-day-old in-vitro grown seedlings produced transgenic calli on B(5) basal medium supplemented with 5 × 10(−6) M Benzlaminopurine (BAP), 2.5 × 10(−6) M each of 2,4-Dichlorophenoxyacetic Acid (2,4-D) and 1-Napthaleneacetic acid (NAA) and 50 mg l(−1) kanamycin after co-cultivation with *Agrobacterium tumefaciens* strains, LBA4404 (pTOK233), EHA105 (pBin9GusInt) and C58C1 (pIG121Hm) all containing beta-glucuronidase (gusA) and neomycin phosphotransferase II (nptII) marker genes at a frequency of 0.9% (Jaiwal et al. 2001). However, we in our study for the first time, transformed green shoots showing strong GUS activity regenerated directly from cotyledonary node explants cultured after co-cultivation with LBA4404 (pTOK233) on B(5) medium containing 6-benzylaminopurine (5 × 10(−7) M) and 100 mg l(−1) kanamycin. Molecular analysis of putative transformed plants revealed the integration and expression of transgenes in T(0) plants and their seeds. Transformation efficiency observed was 4.2% (Table 1).

**Table 1:** **Transformation efficiency expressed in percentage equal to number of explants regenerated and showing PCR amplification/Total number of explants infected × 100**

No. of explants infected	No. of explants regenerated on kanamycin	No. of hardened plants showing PCR amplification	Transformation efficiency (%)
700	200	30	4.2

Various parameters were optimized to establish a reproducible and efficient transformation protocol using at least 10 cotyledonary node explants in three replications for each experiment. The success of transformation was confirmed by transient and stable GUS expression as well as PCR using kanamycin and gene specific primers in transformed explants and regenerated putative plants. Axillary cells of the cotyledonary node explants are known to possess cells that are competent for regeneration and targeted gene delivery (Chandra and Pental 2003). Transformation of cotyledonary nodes leading to the recovery of transgenic plants has been reported earlier in mungbean (Jaiwal et al. 2001).

### Optimizing kanamycin concentration to select transformants

**Figure 1:**
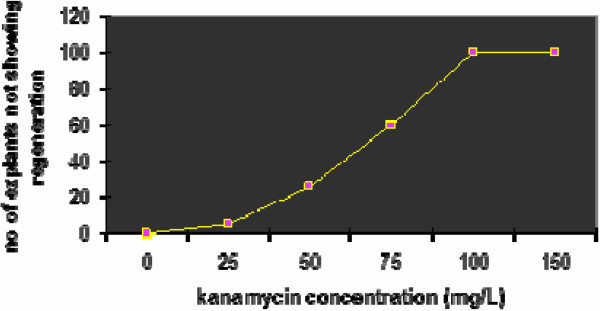
**Kanamycin kill curve.**

Prior to transformation, an effective concentration of antibiotic for the selection of transformed cells was determined by culturing DCN explants on Murashige and Skoog with B5 vitamins (MSB_5_) media containing various concentrations of kanamycin (0, 25, 50, 75, 100, 150 mg/l) (Figure 1). The effect of various concentrations of kanamycin on regeneration response is described below (Table 2). Kanamycin over a concentration of 100 mg/l was observed to be effective to select the transformants derived from DCN explants and caused complete necrosis of the untransformed explants within three weeks and thus was successfully used for selection of transformants. Hence kanamycin at a concentration of 100 mg/l was used for transformants. Earlier Phogat et al. (1999) selected transformed calli of *Vigna radiata* on 100 mg/l of kanamycin concentration.

**Table 2:** **Observed effects of different concentrations of kanamycin on DCN explant**

Kanamycin conc (mg/L)	Regeneration response
0	Green shoots regenerated
25	Green shoots regenerated
50	Green shoots regenerated
75	Pale green shoots were found
100	No shoots observed
150	Explants were bleached

### Factors effecting genetic transformation

Experiments were designed to work out the most optimal conditions for transformation and carried out with a bacterial concentration of 0.5 O.D. with explants derived from 3-day old seedlings (Yadav et al. 2010a) and varying the parameter under study.

### Effect of optical density (O.D.) of Agrobacterium culture

Explants from 3-day old seedlings were co-cultured with *Agrobacterium* culture of varying optical density (O.D.560) between 0.5-1.5, keeping pH 5.8 and kanamycin concentration 100 mg/l. An O.D. of 0.8 was observed to be the give the best transformation response. This O.D. value might be representing the most active log phase of *Agrobacterium* growth and thus very effective for transformation. Similar results were also reported by Jaiwal et al. (1998,2001). Contrary to present report, a decline in transformation efficiency with increase in bacterial cell density has been reported in blackgram (Saini and Jaiwal 2007) which could be due to variation with regard to plant species, explanted tissue, duration of co-cultivation and mode of regeneration.

### Concentration of bacterial cells

Different concentrations of the bacterial cells tested included 10^6^ to 10^9^ cells cm^-3^, transformation frequency (data not shown) increased with increase in concentration of *Agrobacterium* cells up to 10^8^ cells cm^-3^. Similar results have been obtained in most of the grain legumes earlier (Bean et al. 1997).

### Infection time

Infection time of 10, 15, 20 and 30 min was tested and 15 min was observed to deliver the best response. Injuries inflicted with the help of hypodermic needle, enhanced the frequency of transient GUS expression, at the cotyledonary node attachment site. Wounding of the tissue before co-cultivation allows better bacterial penetration into the tissues facilitating the accessibility of plant cells for *Agrobacterium* or possibly stimulated the production of potent *vir* gene inducers like phenolic substances such as acetosyringone and hydroxyl-acetosyringone (Stachel et al. 1985) and enhanced the cell capability for transformation (Binns and Thomashow 1988). Wounding the plant material has been shown to increase transformation frequency (Bidney et al. 1992). Mechanical injury of the meristematic region probably induces meristem reorganizations promoting formation of large transgenic sectors and enhanced recovery of transformants.

### Co-cultivation time

Co-cultivation duration also affected the transformation efficiency. Extending the co-cultivation time up to three days increased the transient transformation frequency and subsequent increase in co-cultivation time decreased the transformation frequency resulting in bacterial over growth. Co-cultivation period of 2 d has been found to be optimum in *Antirrhinum majus* (Holford et al. 1992), *Vigna unguiculata* (Muthukumar et al. 1996), *Vigna radiata* (Jaiwal et al. 2001), *Cajanus cajan* (Mohan and Krishnamurthy 2003), *Glycine max* (Li et al. 2004) and *Nicotiana tabacum* (Uranbey et al. 2007).

### Age of explants

Explants excised from 2- and 3- day-old seedlings were tested for their suitability to achieve higher transformation. Study indicated 3-day old seedlings produced best results. This could be due to differences in the regenerative capacity of the two explants, which in turn is regulated by levels of endogenous hormones.

### GUS analysis

**Figure 2:**
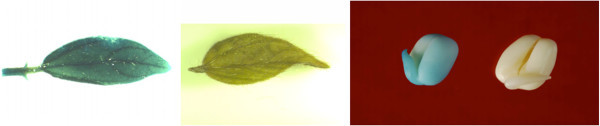
**Stable GUS activity in T**_**0**_**leaf T**_**1**_**seedling (transformed & control).**

To compare transient and stable T-DNA transformation, GUS analysis was done in explants immediately after co-cultivation as well as in young growing tissues of T_0_ and T_1_ generations of putative transformants. Transient GUS expression was not consistent, however, stable GUS expression was observed in young T_0_ leaves and germinating T_1_ seeds (Figure 2). No GUS activity was observed in non-inoculated DCN explants. Explants without pre-culture and mechanical injuries, at the site of regeneration, upon co-cultivation with *Agrobacterium* showed practically very faint or no staining that too at the epicotyls and hypocotyls cut ends. Whereas wounding without pre-culture resulted in intense transient GUS activity at the wounded regions of the treated DCNs. Pre-cultured explants without mechanical injuries showed no GUS activity at the site of regeneration except at the cut regions.

### Acetosyringone concentration

Different levels acetosyringone concentrations were tested and acetosyringone at 100 μM was found to give the best regeneration response. Transformation studies in other plant species have indicated that acetosyringone at an appropriate concentration enhances efficiency of transformation (Srivastava et al. 2009).

### Infection medium

Of the various infection media tested, MSB_5_ gave the highest regeneration response. Other important factors affecting transformation efficiency studied included pre-culture of explants on regeneration medium prior to inoculation. Pre-conditioning of explants for a period of 2–3 days gave the best results. Pre-culture of explants on regeneration medium prior to inoculation and co-cultivation with *Agrobacterium* has been reported to enhance efficiency of transformation in *Vigna unguiculata* (Muthukumar et al. 1996) and *Cajanus cajan* (Geetha et al. 1999). These optimized transformation conditions were used for development of transgenics in greengram. Selection of regenerants was carried out on kanamycin medium combined with previously standardized regeneration procedure.

### Regeneration of transgenic plants

Explants after transformation were regenerated on shoot bud induction medium which contained 100 mg/l kanamycin and 250 mg/l cefotaxime. To achieve the best shoot proliferation response, the concentration of kanamycin was reduced to half at second sub-culture stage. Rooting (90%) was achieved successfully on ½ MSB_5_ medium. The entire cycle was completed in about 80 days. Rooted transgenic plantlets were efficiently hardened and upon transfer to pots attained sexual maturity and produced viable seeds.

### PCR verification

**Figure 3:**
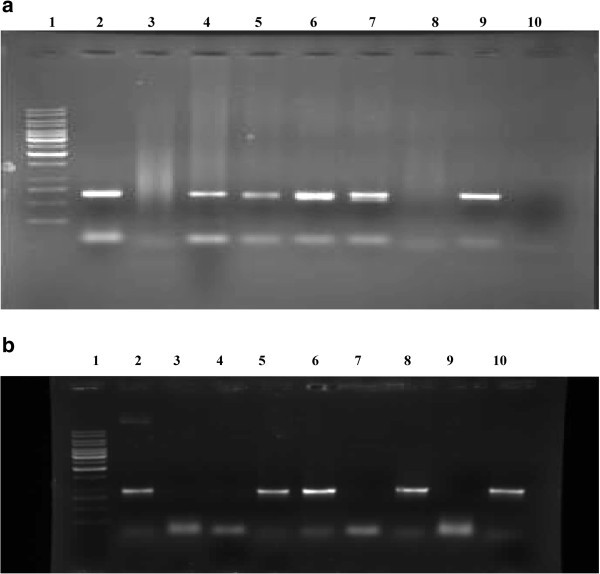
(**a**) **PCR verification of T**_**0**_**putative transgenics in greengram using*npt II*primers.** Lane 1: MW marker, 2: Prositive Control, 3: Negative Control, 4-10: Putative transgenic plants. (**b**) PCR verification of T_0_ transgenics in greengram using gene specific primers. Lane 1: MW marker, 2: Positive control, 3: Negative control, 4-10: Putative transgenic lines.

**Figure 4:**
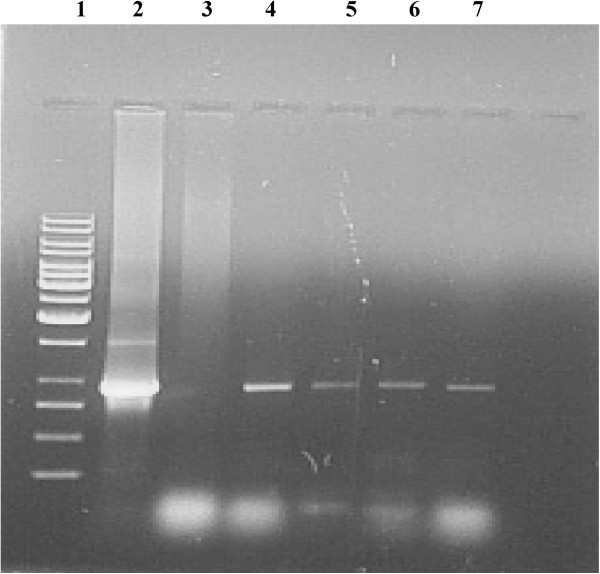
**PCR verification of T**_**1**_**transgenics in greengram using gene specific primers.** Lane 1: MW marker, 2: Positive control, 3: Negative control, 4-7: Putative transgenic lines.

The putative transformants (T_0_) were subjected to PCR with *nptII* and *annexin* gene specific primers to identify stably transformed progeny that survived kanamycin treatment. Genomic DNA when analyzed by PCR using *nptII* and *annexin* gene specific primers yielded 645 and 941 bp DNA fragments corresponding to the coding regions of respective genes (Figure 3a & b). Amplification of 645/941 bp product with *nptII*/*annexin* gene specific primers confirmed transgene integration. PCR positive plants with both *nptII*/*annexin* gene specific primers only were taken to maturity and T_0_ seed was collected which was sown to raise the T_1_ plants under containment. Genomic DNA from T_1_ plants was again analyzed by PCR using *annexin* gene specific primers (Figure 4).

### Confirmation of putative transformants by Southern Blotting

**Figure 5:**
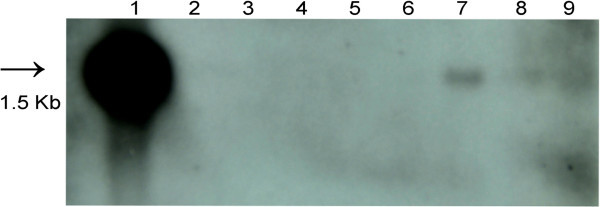
**Southern blot hybridization of the T**_**0**_**putative*annexin*transgenic greengram plants.** Leaf genomic DNA was digested with *Pst I*, separated by electrophoresis on a 0.8% gel, blotted onto Hybond N + membrane and probed with a 941 by *annexin* sequence. Lane 1: Positive control (pCAMBIA 2301 + *annexin* digested with *Pst I* to release 1.5 kb fragment; Lane 2-3: Empty; Lane 4: Negative control; Lane 5-9: T_0_ transformants in which events 7-9 were positive.

Detection of hybridizing bands corresponding to the positive control in at least three of the five putative samples tested confirmed transgene integration (Figure 5). Absence of any hybridization signal in untransformed controls indicated that these plants had no inserted transgene. These results indicate very clearly integration of the transgene in the samples tested and hence the success of transformation protocol developed.

It is summarized that the pre-cultured and mechanically injured three day old DCN explants of *Vigna radiata* were successfully transformed by using bacterial concentration 10^8^ cells cm^-3^. The protocol opens window to genetically enhance the greengram genotypes with genes of proven agronomic importance to attain sustainable production under fragile crop growing environments. Direct shoot regeneration method described here also minimizes the possibility of somaclonal variation besides the applicability of method is season independent by way of using seedling explants. The *annexin* transgenics of greengram developed are being evaluated for physiological and biochemical traits for improved drought stress tolerance.

## Methods

Seeds of a popular cultivar of greengram, ML 267 were obtained from Agricultural Research Station, Lam, and Andhra Pradesh, India. Healthy and uniform seeds were surface sterilized and double cotyledonary node (DCN) explants excised and cultured for efficient shoot regeneration as described by Yadav et al. 2010a.

### Agrobacterium strain and gene construct

**Figure 6:**
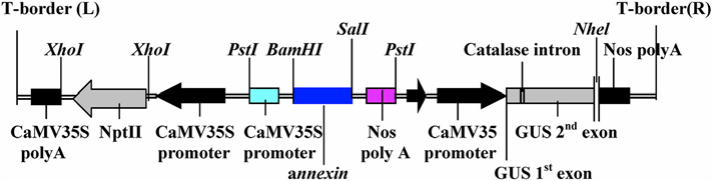
**Schematic representation of T-DNA region of pCAMBIA 2301 having*annexin*gene (6345bp).**

The disarmed *Agrobacterium* strain LBA4404 was used for the genetic transformation of greengram. The gene construct contained a binary vector pCAMBIA 2301 which had β-glucuronidase (GUS) reporter (*uidA*) gene, a neomycin phosphotransferase (*nptII*) gene as plant selection marker driven by cauliflower mosaic virus (CaMV) 35S promoter. The *uidA* gene contains an intron in the coding region to ensure that the observed GUS activity occurred in the plant cell and not due to residual *Agrobacterium cells. Annexin1bj* gene cassette (−*35S promoter-annexin-nos terminater*-) of 1.5 kb in size was cloned in pCAMBIA 2301 vector at *Pst I* site (Figure 6). The recombined vector was transferred into *E. coli* (DH5α) by heat- shock method and finally transferred into *Agrobacterium tumefaciens* strain LBA 4404 by freeze-thaw method. Plasmid DNA isolated from *E. coli* cell upon digestion with *Pst I* resulted in release of 1.5 Kb *annexin* gene cassette. The transformed *Agrobacterium* strain LBA 4404 was used for genetic transformation of DCN explants of greengram.

**Figure 7:**
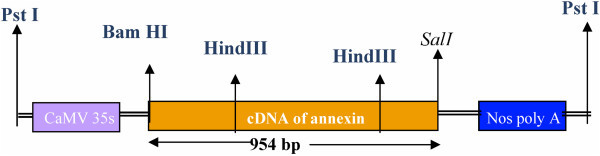
**Restriction enzyme sites in*annexin1bj*gene cassetter (1.5Kb).**

The cDNA of a*nnexin1bj* gene was 954 bp length fragments, it had *Bam HI* restriction site at 5' end and *Sal I* site at 3' end. *Annexin* cDNA also had two restriction sites for *Hind III* enzyme resulting in 3 fragments of 368, 439 and 147 bp lengths (Figure 7). *Npt II* and a*nnexin1bj* gene specific primers were designed which gave 645 and 941 bp product, respectively. *Agrobacterium* strain was grown over night at 28°C in YEB medium containing either 50 mg/l kanamycin. Bacterial cells were pelleted and resuspended in liquid shoot regeneration medium for further use in standardization of various transformation parameters.

### Kanamycin kill curve for selection of transformants

Prior to transformation, an effective concentration of kanamycin was determined for selection of transformants by culturing untransformed DCN explants on shoot bud induction medium containing various concentrations of kanamycin (0–150 mg/l).

### Explant preparation and optimization of conditions for transformation

Selected seeds were rinsed with 70% alcohol for 2 min and the surface sterilized with 0.2% aqueous solution of HgCl_2_ (w/v) for 5 min. The seeds were subsequently washed several times with sterile distill water and cultured on MSB_5_ medium. The best conditions included three-day old DCN explants pre-cultured for 2 days and injured with fine needle at the axillary meristematic region. Over night grown cultures of *Agrobacterium* (0.8A, 1000 μl) were added to the flask containing infection medium and swirled well. Injured explants were added to the infection medium and swirled for 15 min. Infected explants were kept on co-cultivation medium containing 50, 100 and 200 μM acetosyringone for 2, 3 and 4 days. After co-cultivation explants were washed with cefotaxime (250 mg/l) and cultured on shoot bud induction medium containing 100 mg/l kanamycin as selection agent. Explants were sub-cultured onto fresh medium every 15 days.

### Transformation

The conditions optimized for the best regeneration earlier were practiced to get the finest transformation response to develop transgenic greengram with *annexin 1bj* gene by Agrobacterium mediated approach. The transformation of three day old DCN explants was carried out by using LBA 4404 strain of *Agrobacterium tumefaciens* harbouring pCAMBIA 2301 binary vector containing *annexin 1bj* gene under the control of CaMV 35S promoter. Experiments were repeated on a regular interval to generate more of independent events for selecting the promising transgenic plants of green gram. Untransformed explants were kept as regeneration control on kanamycin free media.

### Selection of putative transgenics

The transformants were selected on 100 mg/l kanamycin in shoot bud induction medium (MS B_5_ containing BAP and NAA) for first 30 days of culture. Subsequently, the kanamycin concentration was reduced to 50 mg/l for next cycle of 30 days in shoot elongation and proliferation medium (MS B_5_ containing reduced levels of BAP and NAA). The regenerated shoots were rooted on ½ MS B_5_ medium and were taken to maturity in a transgenic glass house after primary hardening.

### GUS histochemical analysis

Transient and stable histochemical GUS assay was carried out in different tissues essentially as described by Jefferson (1987).

### PCR Analysis of putative transformants

The leaf genomic DNA from T_0_ and T_1_ plants was isolated by Cetyl Trimethyl Ammonium Bromide (CTAB) method and used for molecular characterization of putative transgenics by PCR using *nptII* and *annexin* gene specific primers. T_0_ plants were analyzed by PCR using *npt II*/*annexin* gene specific primers while T_1_ plants were analyzed using *annexin* gene specific primers only. The sequence of oligonucleotide for *npt II* primers was, Forward: 5^’^ - AAT ATC ACG GGT AGC CAA CG – 3^’^; Reverse: 5^’^ - GCT TGG GTG GAG AGG CTA TT - 3’ and *annexin* gene specific primers was, Forward: 5^’^- ATG GCG ACT CTT AAG GTT TCT T –3^’^; Reverse: 5^’^ - TCA CCG AGA AGT GCG ATG AG– 3’. PCR was carried out with 60 ng of purified genomic DNA, and Dream *Taq* polymerase (Genetix) in a Applied Biosystems thermal cycler with previously standardized run conditions which included initial denaturation at 94°C for 5 min followed by 30 cycles of 94°C for 1 min, 55°C for 1 min and 72°C for 30 s and final extension at 72°C for 5 min. Plants confirming positive with PCR were taken to maturity and their seed was collected. The T_0_ seed so collected was sown in pots to raise T_1_ plants. The leaf genomic DNA from the T_1_ plants was analyzed by PCR using *annexin* gene specific primers. The genomic DNA from the untransformed control plants and pCAMBIA 2301 *annexin* were used as negative and positive controls, respectively. The amplified products were separated by electrophoresis on a 0.8% agarose gel and visualized with ethidium bromide.

### Southern Blot Hybridization

Leaf genomic DNA was isolated by CTAB method from the putative T_0_*annexin* greengram transgenics developed by *Agrobacterium* mediated transformation. Integration of foreign gene in the host genome was determined by Southern analysis as per procedure described by Sambrook et al. 1989. Genomic DNA was digested with *Pst I* restriction enzyme which releases 1.5 kb gene cassette containing 954 bp *annexin* gene. The restricted DNA was blotted onto a Hybond N + membrane. Probe DNA was prepared from the PCR amplified product (941 bp) of *annexin* gene. The probe was made hot as per standard procedure with ^32^P. The Hybond N + membrane was incubated with pre-hybridization solution for 4 hrs and hybridization solution (containing hot *annexin* 941 bp probe DNA) for 20 hrs.

The Hybond N + membrane was washed and dried. Then the Hybond N + membrane was exposed to autoradiography film for two weeks. DNA isolated from an untransformed control plant was also tested for the presence of *annexin* gene in order to determine if transgene was present.

## Abbreviations

DCN: Double Cotyledonary Node
GUS: β-glucuronidase
PCR: Polymerase Chain Reaction
BAP: Benzlaminopurine
MSB5: Murashige and Skoog with B5 vitamins
CaMV: Cauliflower Mosaic Virus (CaMV)
CTAB: Cetyl Trimethyl Ammonium Bromide.
